# Regional biaxial mechanical data of the mitral and tricuspid valve anterior leaflets

**DOI:** 10.1016/j.dib.2019.103961

**Published:** 2019-05-22

**Authors:** Devin Laurence, Colton Ross, Samuel Jett, Cortland Johns, Allyson Echols, Ryan Baumwart, Rheal Towner, Jun Liao, Pietro Bajona, Yi Wu, Chung-Hao Lee

**Affiliations:** a Biomechanics and Biomaterials Design Laboratory (BBDL), School of Aerospace and Mechanical Engineering, The University of Oklahoma, Norman, OK 73019, USA; b Center for Veterinary Health Sciences, Oklahoma State University, Stillwater, OK 74078, USA; c Advanced Magnetic Resonance Center, MS 60, Oklahoma Medical Research Foundation, Oklahoma City, OK 73104, USA; d Department of Bioengineering, The University of Texas at Arlington, Arlington, TX 76019, USA; e Department of Cardiovascular and Thoracic Surgery, The University of Texas Southwestern Medical Center, Dallas, TX 75390, USA; f Institute for Biomedical Engineering, Science and Technology (IBEST), The University of Oklahoma, Norman, OK 73019, USA

**Keywords:** Biaxial mechanical testing, Regionally-varied mechanical properties, Atrioventricular heart valve biomechanics

## Abstract

The collective data associated with this article presents the biaxial mechanical behavior for six smaller, delimited regions of the mitral valve and tricuspid valve anterior leaflets. Each data set consists of five columns of data, specifically: (i) biaxial testing protocol ID, (ii) circumferential stretch, (iii) radial stretch, (iv) circumferential membrane tension, and (v) radial membrane tension. For further elaboration regarding methodologies or results of the biaxial mechanical characterization please refer to the companion article Laurence, 2019.

**Table:** Specifications Table

Subject area	*Mechanical Engineering*
More specific subject area	*Biomechanics of Biological Materials*
Type of data	*Text Files (.txt)*
How the data was acquired	*Biaxial Mechanical Testing*
Data format	*Analyzed*
Experimental factors	*Leaflet type (MVAL: mitral valve anterior leaflet, TVAL: tricuspid valve leaflet), leaflet region (A, B, C, D, E, F), tissue direction (circumferential and radial), tissue loading rate (2.*29 N/min*), testing temperature (37°C)*
Experimental features	*Employed biaxial testing methods to characterize the anisotropic mechanical responses of six tissue regions of the MVAL and TVAL*
Data source location	*Norman, OK, United States*
Data accessibility	*All data are presented along with this article.*
Related research article	*D. Laurence, et al., An investigation of regional variations in the biaxial mechanical properties and stress relaxation behaviors of porcine atrioventricular heart valve leaflets, J. Biomech. 83 (23), 2019, 16–27.*

**Table:** 

**Value of the data** • Quantification of regional variances in the mechanical behavior of the mitral and tricuspid valve anterior leaflets. • Refinement of computational models to consider leaflet tissue regional mechanical heterogeneities. • Reference for the development of heart valve repair and replacement therapeutics.

## 1 Data

**Fig. 1:**
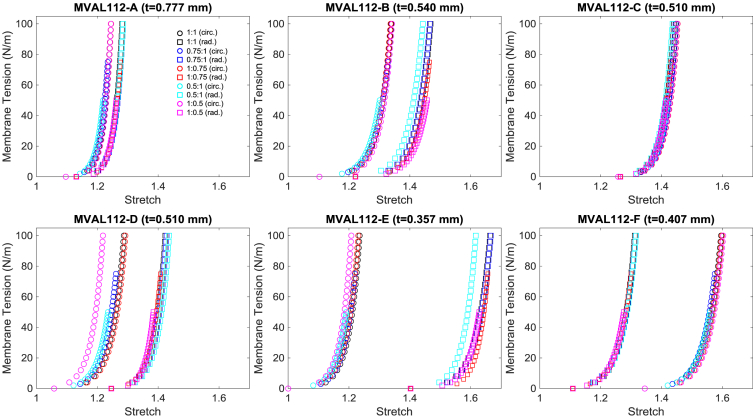
First example data (MVAL112) of membrane tension versus stretch mechanical responses for the six MVAL regions under all five loading protocols. *(Every fourth data point was plotted for visualization purposes.) t* denotes the tissue specimen's thickness used in calculation of the first-PK stress.

**Fig. 2:**
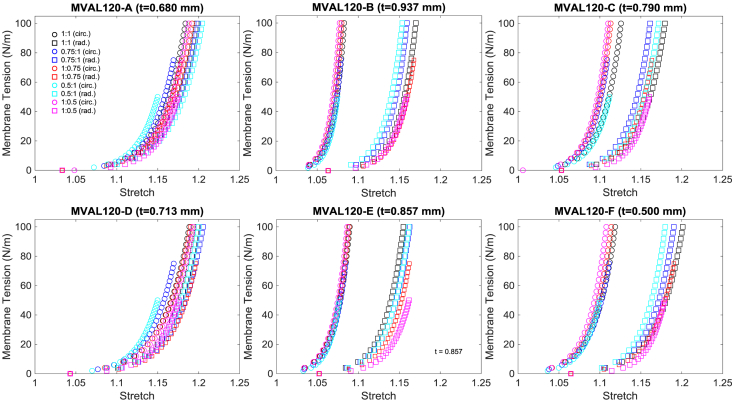
Second example data (MVAL120) of membrane tension versus stretch mechanical responses for the six MVAL regions under all five loading protocols. *(Every fourth data point was plotted for visualization purposes.) t* denotes the tissue specimen's thickness used in calculation of the first-PK stress.

**Fig. 3:**
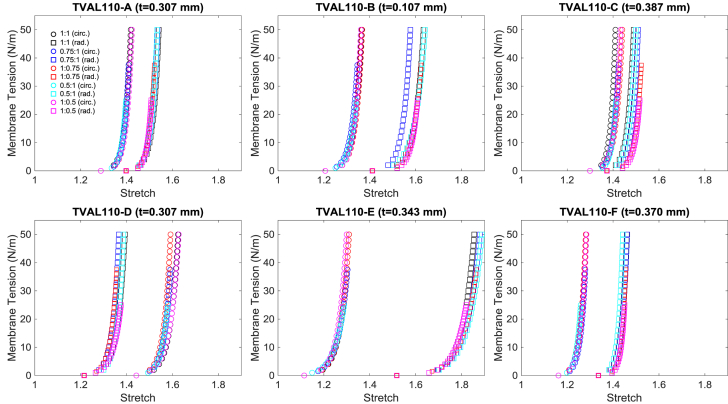
First example data (TVAL110) of membrane tension versus stretch mechanical responses for the six TVAL regions under all five loading protocols. *(Every other data point was plotted for visualization purposes.) t* denotes the tissue specimen's thickness used in calculation of the first-PK stress.

**Fig. 4:**
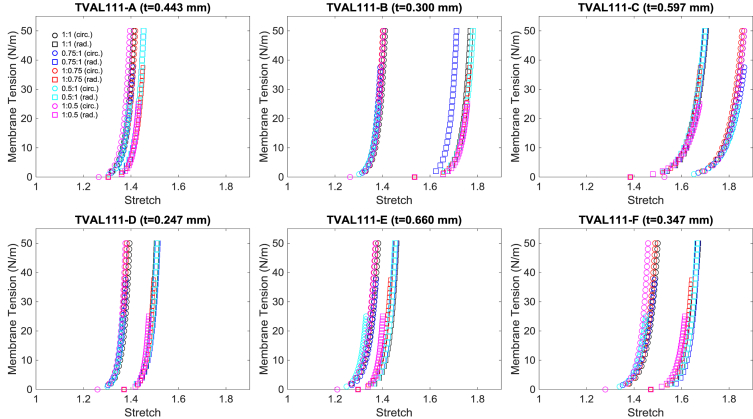
Second example data (TVAL111) of membrane tension versus stretch mechanical responses for the six TVAL regions under all five loading protocols. *(Every other data point was plotted for visualization purposes.) t* denotes the tissue specimen's thickness used in calculation of the first-PK stress.

The data presented in this document provide the mechanical response of six small regions (A, B, C, D, E, and F) of both the mitral valve anterior leaflet (MVAL) and tricuspid valve anterior leaflet (TVAL). Each data set starts with the specimen's thickness (first row) and the effective specimen size (second row) and follows by five columns of data. The first column provides a value from 1 to 5, which correspond to the loading protocol ID, i.e., *T*_*circ*_:*T*_*rad*_ = 1:1, 0.75:1, 1:0.75, 0.5:1, and 1:0.5, respectively. Here, *T*_*circ*_ and *T*_*rad*_ are the membrane tensions in the circumferential and radial directions, respectively. The second and third columns provide the stretch values with respect to the circumferential and radial directions (*λ*_*circ*_ and *λ*_*rad*_). The fourth and fifth columns provide the membrane tension values (N/m) in the circumferential and radial directions (*T*_*circ*_ and *T*_*rad*_). The collective data consists of 10–13 data sets for each of the six MVAL/TVAL tissue regions. Variations of the number per tissue region may result from the dissected tissues being too small for testing, testing failure due to system error(s), or failed mechanical testing owing to tissue tearing. Two sample sets of data for all six tissue regions of the MVAL and TVAL [1] are provided in Fig. 1, Fig. 2 and in Fig. 3, Fig. 4, respectively.

## 2 Experimental design, materials, and methods

### 2.1 Tissue retrieval and storage

Porcine hearts were obtained from a local FDA-approved slaughterhouse (Country Home Meats, Edmond, OK), transported to the laboratory, and cleaned of blood clots before being stored in a standard freezer at −14 °C [2], [3], [4].

### 2.2 Tissue dissection and segmentation

**Fig. 5:**
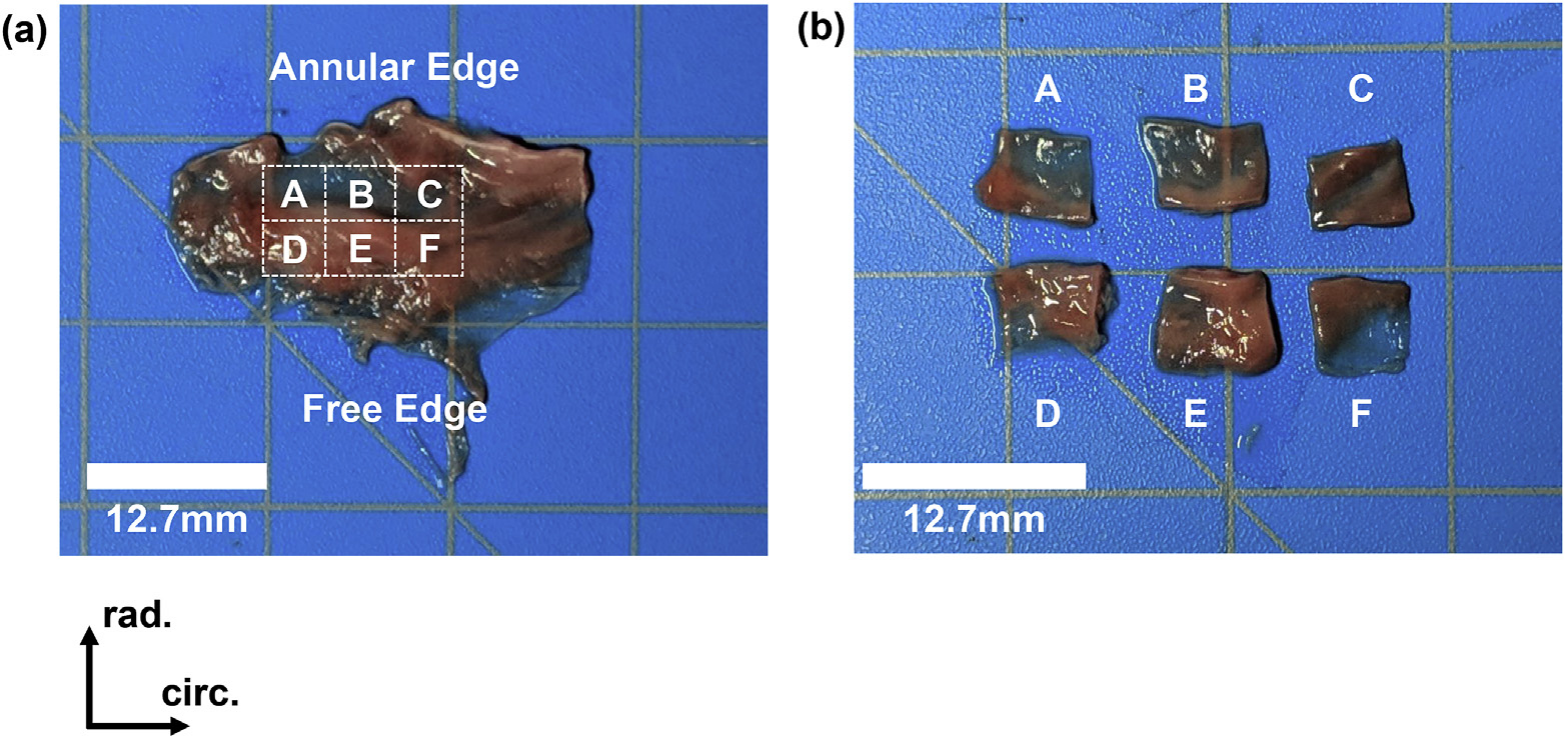
Experimental photos for demonstrating the regional segmentation process from (a) the whole leaflet (the TVAL as shown) to (b) the six smaller segmented regions used for testing.

For dissection, the hearts were slowly thawed in a bath of warm water and dissected to retrieve the MVAL and TVAL tissues (Fig. 5a). Each leaflet was then segmented into six smaller regions of a 6  × 6 mm dimension (Fig. 5b) to quantify the regional variations in the tissue's mechanical properties [1]. The dissected tissue regions were properly labelled with the appropriate tissue directions, placed in a labelled container of phosphate buffered saline (PBS), and stored in a refrigerator at 4 °C until testing within two days [5].

### 2.3 Tissue mounting to the biaxial mechanical testing apparatus

For biaxial mechanical testing, the 6 × 6mm tissues were mounted to a commercial biaxial testing system (BioTester, CellScale, Waterloo, ON, Canada) to create an effective testing region of 3.5 × 3.5mm. Care was taken to ensure the principal tissue directions (i.e., circumferential and radial directions) aligned with the axes of the testing system (i.e., *X*- and *Y*-directions). Then, a square array of fiducial markers was applied to the central one-third of the mounted tissue using a surgical pen for optical-based strain calculations. The tissue was submerged in a bath of PBS at 37 °C and subjected to biaxial mechanical testing as discussed in the next subsection.

### 2.4 Biaxial mechanical testing

The biaxial testing consisted of an equi-biaxial preconditioning protocol to exercise the tissue to its *in vivo* state, and five testing protocols with loading ratios of *T*_*circ*_:*T*_*rad*_ = 1:1, 0.75:1, 1:0.75, 0.5:1, and 1:0.5. The maximum membrane tension values of 100 N/m for the MVAL and 50 N/m for the TVAL were chosen based on the previous investigations [6], [7]. Each protocol consisted of eight repeated loading/unloading cycles with data collected from the load cells and high-resolution CCD camera at a rate of 15 Hz. The data from the last loading cycle was used in subsequent stress and strain calculations as described in the next subsection.

### 2.5 Tissue stress and strain calculations

First, the images from the last cycle of each protocol were tracked using the data image correlation methods of the testing system's software to provide the time dependent locations of the four fiducial markers. Then, the fiducial markers were treated as a four-node bilinear finite element to compute the deformation gradient **F** using [8], [9], [10].

(1) $$F=F\left(X,t\right)=\frac{\partial x\left(X,t\right)}{\partial X}=\left[\begin{array}{cc} \sum_{I=1}^{4}B_{XI}\left(X\right)u_{I}\left(t\right) & \sum_{I=1}^{4}B_{YI}\left(X\right)u_{I}\left(t\right)\\ \sum_{I=1}^{4}B_{XI}\left(X\right)v_{I}\left(t\right) & \sum_{I=1}^{4}B_{YI}\left(X\right)v_{I}\left(t\right) \end{array}\right]\text{.}$$

Here, the *B*_*XI*_'s and *B*_*YI*_'s are the shape function derivatives for node (marker) *I* in the *X*- and *Y*-directions, respectively, and the *u*_*I*_'s and *v*_*I*_'s are the corresponding nodal (marker) displacements. The deformation gradient was then used to compute the right Cauchy-Green deformation tensor **C** and Green-Lagrangian strain tensor **E** by

(2) $$C=F^{T}F\;\text{and}\;E=\frac{1}{2}\left(C-I\right)\text{,}$$

where **I** is the second-order identity tensor. The stretch values in the testing directions (*λ*_*circ*_ and *λ*_*rad*_) were calculated by taking the square roots of the principle values of **C**.

The corresponding membrane tension values (*T*_*circ*_ and *T*_*rad*_) were calculated using the load cell force readings and the effective testing edge length of 3.5 mm:

(3) $$diag\left[T_{circ},T_{rad}\right]=\frac{1}{L}diag\left[f_{circ},f_{rad}\right]\text{,}$$

where *f*_*circ*_ and *f*_*rad*_ are the applied forces in the circumferential (*X*) and the radial (**Y**) directions, respectively, and *L* is the effective testing edge length. For comparisons of this data to other stress values, the membrane tension values can be converted to the 1st-Piola Kirchhoff stress tensor **P**, the 2nd-Piola Kirchhoff stress tensor **S**, or the Cauchy stress tensor **σ** using

(4) $$P=\frac{1}{t}\left[\begin{array}{cc} T_{circ} & 0\\ 0 & T_{rad} \end{array}\right]\text{,}S=F^{-1}P\text{,and}\;\sigma =J^{-1}{\mathrm{PF}}^{T}.$$

Here, *t* is the tissue thickness and *J* is the Jacobian of the deformation tensor **F**.
