# Supplementary material for: Regional biaxial mechanical data of the mitral and tricuspid valve anterior leaflets
Source: Data Brief. 2019 May 22;24:103961. doi: 10.1016/j.dib.2019.103961 (PMC6545396; doi:10.1016/j.dib.2019.103961)
Supplement: Multimedia component 1 [file mmc1.docx]

**Declarations of Interest Statement**

We hereby declare that there is no duplicate publication elsewhere of any part of this work. There are no commercial relationships which might lead to a conflict of interests. The typescript has been read and agreed by all authors.

We hereby declare that all authors were fully involved in the study and preparation of the manuscript and the material within has not been and will not be submitted for publication elsewhere.

Authors:

Devin Laurence

Colton Ross

Samuel Jett

Cortland Johns

Allyson Echols

Ryan Baumwart

Rheal Towner

Jun Liao

Pietro Bajona

Yi Wu

Chung-Hao Lee

For correspondence:

Chung-Hao Lee, Ph.D.

Assistant Professor

School of Aerospace and Mechanical Engineering

Affiliated Faculty Member

Institute for Biomedical Engineering, Science, and Technology

The University of Oklahoma

865 Asp Ave., Felgar Hall Rm. 219C

Norman OK 73019-3609

email: [ch.lee@ou.edu](mailto:ch.lee@ou.edu)

Tel: 405-325-4842
